# Prevalence of Human Polyomavirus JC and BK in Normal Population

**DOI:** 10.31557/APJCP.2020.21.10.2877

**Published:** 2020-10

**Authors:** Lila Karimi Dehcheshmeh, Manoochehr Makvandi, Ali Timori

**Affiliations:** 1 *Infectious and Tropical Diseases Research Center, Health Research Institute, Ahvaz Jundishapur University of Medical Sciences, Ahvaz, Iran. *; 2 *Virology Department, School of Medicine, Ahvaz Jundishapur University of Medical Sciences, Ahvaz, Iran. *

**Keywords:** JC virus, BK virus, polymerase chain reaction

## Abstract

JC virus (JCV) , and BK virus (BKV) can remain latency in kidney and excrete via urine asymptomatically. JCV has been associated with colorectal and bladder cancers. BKV has been linked with lung, pancreas, liver, urogenital tract, head and neck cancers. Therefore, the frequency of JCV DNA and BKV DNA are essential to evaluate in urine samples of healthy individuals.

**Materials and Methods:**

Hundred sixty four urine samples were collected from healthy subjects [96 females and 68 males]. DNA was extracted and detection of JCV DNA and BKV DNA was carried out by PCR . The analysis of sequencing and construction of phylogenetic tree were performed for the samples positive for JCV DNA and BKV DNA.

**Results:**

Ten (6.09%) urine samples [5/96(5.2%) females and 5/68( 8.82) males] were tested positive for JCV DNA (P= 0.814). The results of sequencing and phylogenetic tree showed the isolated JCV DNA were cluster with 3A genotype. 21/164 (12.8%) samples were tested positive for BKV DNA [11/96(11.45%) females and males 10/68(14.7%)] ( P= 0.63). The results of sequencing and phylogenetic tree showed that the isolated BKV was cluster with genotype III.

**Conclusion:**

In the present study 6.09% and 12.8% of the healthy individuals showed positive for JCV DNA (genotype 3A) and BKV DNA(genotype III) respectively. With regard to life threating diseases by BKV and JCV in immunocomprsied patients , the screening BKV DNA and JCV DNA should be implemented for patients with cancer /autoimmune diseases /organ recipient/ multiple sclerosis (MS), prior to immunosuppression therapy or immunomodulatory agents treatment.

## Introduction

JCV or John Cunningham virus (JCPyV) and BK Polyomavirus (BKPyV) are belong to polyomaviridae family (DeCaprioet al.,2013). They are small, non-enveloped viruses with an icosahedral capsid containing a double stranded DNA genome of ~ 5.2 kbp. JCV and BKV are prevalent in human populations worldwide (Moens et al.,2017, Boothpur et al., 2010). Previous studies have indicated that 70% to 80% of adult population were seropositive against JCV and BKV (Nali et al., 2012). JCV was excreted in the urine of > 40% of human population above 30 years old (Jeong et al., 2004). BKV virus has been detected in urine sample of 10%-60% of the kidney transplant patients (Garces et al., 2010). Both the viruses share same rout of transmission and are transmitted via respiratory or oral-fecal rout (Bofill-Mas et al., 2001). Genetic variations of JCV and BKV are useful information in understanding of the genetic evolution of these viruses and history of human immigration. Based on hypervariable region of VP1, the JCV has been classified into 7 genotypes (DeCaprioet al., 2013; Moens et al., 2017; Boothpur et al., 2010; Nali et al., 2012; Jeong et al., 2004; Garces et al., 2010; Bofill-Mas et al., 2001). The JCV genotype one is dominant in Europe, genotypes 2 and 7 are dominant in Asia, and type 3 and 6 are dominant in Africa, while genotype 4 is found in Europe and United States (DeCaprioet al., 2013; Nali et al., 2012; Jeong et al., 2004; Garces et al., 2010). Based on hypervariable region of VP1 , the BKV has been classified into 4 genotypes (DeCaprioet al., 2013). BKV genotype I is the most prevalent in different regions of the worldwide (~ 80%), followed by genotype IV (~ 15%) which mainly found in Europe and East Asia. Genotypes II and III are rare (~ 5%) in all geographic regions (Morel et al., 2017). 

Both JCV and BKV infect human in early childhood without any symptom and have ability to persist and remain latent in organs such Kidney, Brain, and B lymphocytes (Lazrek et al.,2011; Stolt et al., 2003). JCV has been associated with cognitive decline, dementia, strokes, and brain tumors (Katona et al., 2009). 

JCV has been associated with diseases such as encephalitis and nephropathy (Drachenberg et al 2007; Tan et al., 2010). Reactivation of the JCV occurs under prolonged immunosuppression condition which may result in a fetal disease, Progressive multifocal leukoencephalopathy (PML) (Agostini et al., 1997). The JCV DNA has been detected in various neoplastic lesions such as oligodendroglioma, astrocytoma medulloblastoma, ependymoma, glioblastoma, colorectal carcinoma, gastrointestinal and bladder cancers (Hirsch et al.,2013: Pinto et al., 2014: Elena et al., 2015: Maryam et al., 2018: Casini et al., 2005: Robles et al., 2013). 

BKV has been associated with hemorrhagic cystitis, interstitial nephropathy, and neurological impairment in immunocompromised (Nali et al., 2012; Lazrek et al., 2011).

In animals BKV frequently develops ependymomas, pancreatic islet tumors, osteosarcomas, fibrosarcomas, liposarcomas, osteosarcomas, nephroblastomas and gliomas (Tognon et al., 2003). BKV have been associated with prostate, colorectal and bladder cancers (Elena et al., 2015; Maryam et al., 2018; Casini et al., 2005; Robles et al., 2013).

With regard to consequences of BKV and JCV cause important human diseases and cancers, this study was conducted to evaluate the prevalence of JCV DNA and BKV DNA in urine samples of healthy subjects in Ahvaz city, Iran. 

## Materials and Methods

*Ethic Consent*

The project with Ethic code IR.AJUMS.REC.1395.826 was approved by ethic committee of Ahvaz Jundishapur university of Medical Sciences, Ahvaz Iran. The informed ethic consent was received from all participants in this survey.

*Subjects and samples*

A total of 164 healthy subjects [96(58.53%) females from age 5 to 75 years and 68(41.46%) males from age 5 to 90 years] were selected in Ahvaz city ,Iran during November 2014 to January 2015. The consecutive urine samples were collected and stored in -80 refrigerator until DNA extraction and PCR tests. None of the participants was taking drugs that might influence the urinary excretion, and no pregnant female was selected. 

*DNA extraction*

DNA was extracted from 200 µl of urine sample using the high pure viral nucleic acid kit (Roche, Germany) according to manufacturer’s instructions. The purity and concentration of the DNA of the each sample was checked by NanoDrop spectrophotometer (Thermo Scientific,USA).

*Detection of JCV DNA By PCR*

The following forward primer VP1 (2093-2111) 5-´TTTTGGGACACTAACAGGAGG-3́, and reverse primer VP1- LTAg (2748-2729) 5́-AGCAGAAGACTCTGGACATG-3́ were used for detection of JCV DNA. The PCR reaction mixture containing of 12.5 µl master mix red (Amplicon, Denmark), template DNA 4ng/µl, 1 µl (0.5 µM) of each forward and reverse primers, and distilled water up to 25 µl. The PCR program was initiated by denaturation at 95°C for 5 min, followed by 30 cycles of 95°C for 30 sec, 60°C for 40 sec, 72°C for 40 sec, and a final extension step at 72°C for 5 min. The PCR products were electrophoresed on 2% agarose gel containing safe stain, the expected PCR product was 656 bp (Haghi et al., 2019).

*Detection of BKV DNA By PCR*

The following forward primer BKF VP1 (1618-1637) 5-´ CAAGTGCCAAAACTACTAAT-3́, and reverse primer BKV VP1 (1925-1944) 5́- TGCATGAAGGTTAAGCATGC -3́ were used for detection of BKV DNA. The PCR reaction mixture containing of 12.5 µl master mix red (Amplicon, Denmark), 5 ng/µl template DNA, 1 µl (0.5 µM) of each forward and reverse primers, and distilled water up to 25 µl. the PCR program was started at initial denaturation at 95°C for 5 min, with 30 cycles of 30 sec at 95°C, 30 sec at 56°C, 30 sec at 72°C, and a final extension at 72°C for 10 min. PCR products were subjected to gel electrophoresis in a 2% agarose gel and visualized by UV light exposition. The expected PCR product was 327 bp.( Jin et al.,1993)

*Analysis of JCV and BKV sequencing*

Randomly 2 positive PCR products of JCV DNA and one positive PCR product BKV were selected and sequenced (Bioneer company, South Korea). The sequences were blasted using available databases www.ncbi.gov. The results of sequences of two positive JCV DNA and one positive BKV DNA were submitted to Gene Bank to obtain accession number. 

*Phylogenetic tree analysis for BKPyV and JCPyV virus*

The phylogenetic tree was constructed by Neighbor joining method using VP1 region of BKV and VP1- LTAg region of JCV. The Neighbor joining method was performed with MEGA software version 6, under Kimura two-parameter model, with Bootstrap 1,000 replicates . (Studier et al., 1988). 

*Statistical analysis*

The Chi-square and fisher’s exact tests were used for frequency of JCV and BKV among the gender and age-groups. Values of p<0.05 was considered significant. SPSS 22 software was used for analyzing.

## Results

The results of this study showed that 10/ 164(6.09%) samples [5/96(5.2%) females and 5/68( 7.35% ) males] were tested positive for JCV DNA (P= 0.814). Also 21/164(12.8%) samples were tested positive for BKV DNA [11/96 (11.45%) females and males 10/68(14.7%)] respectively (P= 0.63 ). The frequency of JCV in the age group 60-69 was higher than in age group < 20 years (p=0.035). The rate of BKV in the age group 60-79 was higher than in age group < 20 years (p=0.045) (Table 1 ). 

*Analysis BKV , JCV sequencing and phylogenetic tree*

**Figure 1:**
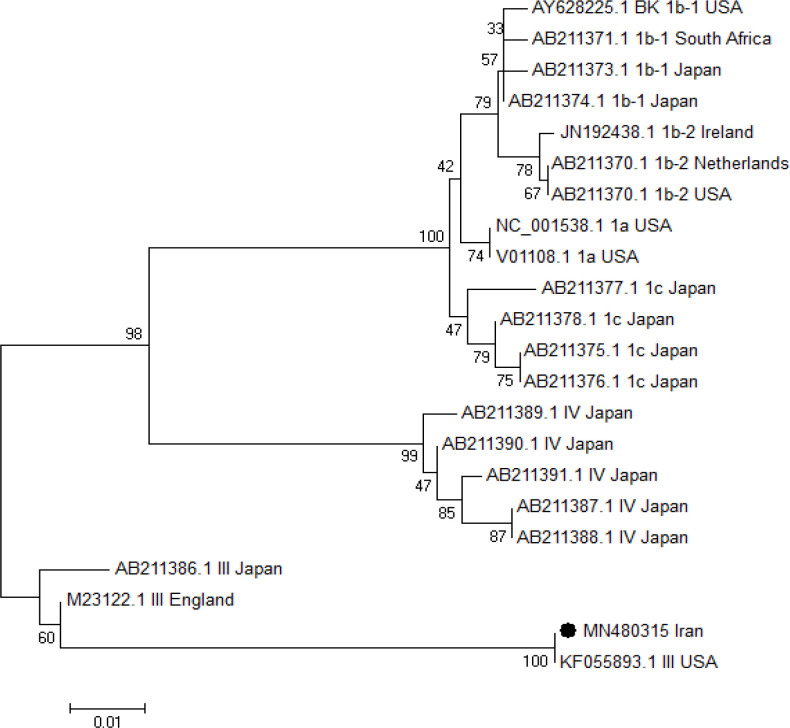
Phylogenetic Tree was Constructed by Neighbor Joining Method for VP1 Region of the BKV DNA Isolated from a Urine Sample. The isolated BKV DNA (MN480315) from Iran was compared with other different isolated BKVs genotypes (I-IV) retrived from Genebank. The isolated BKV (MN480315) in the black circle was cluster with BKV genotype III USA (KF055893.1). The constructed neighbor joining method is under Kimura two-parameter model by 1000 bootstrap replicates. The scale Bar =0.01

**Figure 2:**
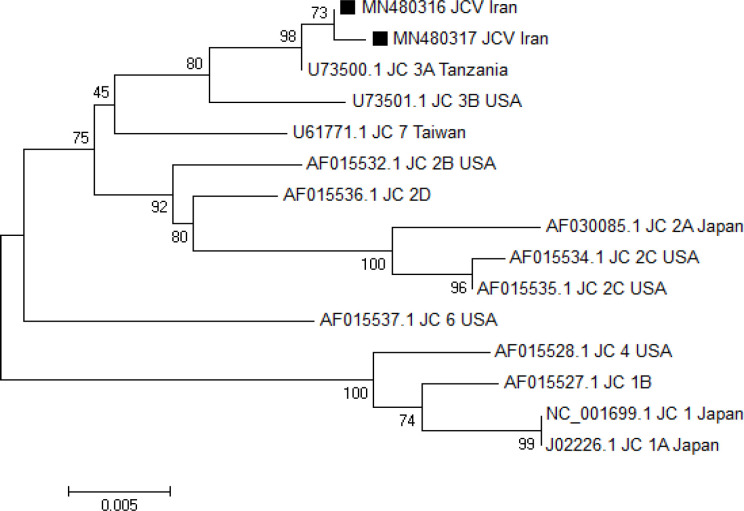
Phylogenetic Tree was Constructed by Neighbor Joining Method for VP1 Region of the Isolated JCV in Ahvaz city, Iran. The two isolated JCV (MN480316 and MN480317). with black squar were compared with different JCVs genotypes(1-4) and subtypes isolated from different regions of the world. The two isolated JCVs were cluster with JCV genotype III Tanzania (U73500). The constructed neighbor joining method is under Kimura two-parameter model by 1000 bootstrap replicates The scale Bar =0.005

The isolated BKV DNA with accession number (MN480315) and two isolated JCV DNA with accession numbers (MN480316 and MN480317) were recorded in GeneBank. The results of sequencing and phylogenetic tree showed that the detected BKV DNA was genotype III and cluster with BKV genotype III ,USA (KF 055893) (Figure 1). The analysis of sequencing and phylogenetic tree of the two detected JCV DNA revealed that both the strains were genotype type III and cluster with genotype 3A isolated from Tanzania (U73500) (Figure 2).

**Table 1:** Frequency of JCV DNA and BKV DNA in Healthy Individuals

Age-group	JCV				*P*-value	BKV				*P*-value
	Male		Female			Male		Female		
	+ve	-ve	+ve	-ve		+ve	-ve	+ve	-ve	
<20	2	16	0	10		0	18	0	10	
20-29	1	6	0	22	0.970	1	6	0	22	0.980
30-39	3	9	3	23	0.490	1	11	2	24	0.230
40-49	0	6	2	11	0.900	0	6	1	12	0.840
50-59	1	8	1	12	0.780	1	8	0	13	0.900
60-69	2	6	4	4	0.035*	0	8	2	6	0.240
70-79	0	4	1	2	0.880	2	2	0	3	0.045*
80-89	1	3	0	1	0.930	0	4	0	1	0.410

Tabbe,1, shows the distributions of JCV DNA and BKV DNA in the age group <20 and compared it with different age groups. The rate of JCV in the age group 60-69 showed higher than in age group < 20 years (p=0.035). Also the rate of BKV in the age group 60-79 showed higher than in age group < 20 years (p=0.045).

## Discussion

BKV and JCV are prevalent in ~ 80% of world population. The detection of BKV in blood of renal graft recipients is regarded as a good marker for determination of active BKV infection (Tetsuya et al., 2005). The presence of JCV DNA in blood of immunocompromised subject may reflect as a risk factor for PML (Castro et al., 2017). The JCV DNA has been detected in various neoplastic lesions such as oligodendroglioma, astrocytoma medulloblastoma, ependymoma, glioblastoma, colorectal carcinoma, gastrointestinal and bladder cancers (Hirsch et al., 2013; Pinto et al., 2014; Elena et al.,2015; Maryam et al 2018; Casini et al.,2005; Robles et al., 2013). 

In animals BKV frequently developed ependymomas, pancreatic islet tumors, osteosarcomas, fibrosarcomas, liposarcomas, osteosarcomas, nephroblastomas and gliomas (Tognon et al., 2003). The BKV has been associated with prostate cancer colorectal and bladder cancers (Elena et al., 2015; Maryam et al., 2018; Casini et al., 2005: Robles et al., 2013).

In our study 10/164 (6.09%) urine samples [5/96(5.2%) females and 5/68( 7.35%) males] were tested positive for JCV DNA (P= 0.814). and 21/164(12.8%) samples were tested positive for BKV DNA [11/96(11.45%) females and males 10/68(14.7%)] ( P= 0.63 ). 

The results of our study showed that the frequency of JCV in the age group 60-69 was higher than in age group < 20 years (p=0.035) and the frequency of BKV in the age group 60-79 was higher than in age group < 20 years (p=0.045). It has been reported the frequency of JCV was found higher in the age group above 40 years old in the Italian general population (Pagani et al., 2003)

The prevalence of JCV DNA and BKV DNA in the urine samples of healthy individuals were reported in different regions of the world. In the Brazilia JCV (17.5%) and BKV (12.5% ), (Machado et al., 2011), Portugal JCV (23.9%) and BKV 1.8% (Rodrigues et al., 2007). Switzerland JCV (19%) and BKV (7%) (Egli et al., 2009), Tunisia of JCV DNA (13%) and BKV DNA (6%) (Hanen et al., 2016). 

The results of sequencing and phylogenetic tree showed that the detected BKV DNA was genotype III and clustered with BKV genotype III ,USA (KF 055893). The analysis of sequencing and phylogenetic tree of the two detected JCV DNA revealed that both the strains were genotype type III and clustered with genotype 3A isolated from Tanzania (U73500).

BKV causes transplant kidney function abnormity or failure; BKV is reactivated when immune function of host becomes weak (Ambalathingal et al., 2017). Thus individuals who receive immunosuppressive therapy, especially renal transplantation recipients, are more likely to have BKV activation and may induce BKV associated nephropathy (BKVAN) (Ambalathingal et al., 2017).

PML is a demyelinating disease of the central nervous system that results from lytic JCV infection of glial cells in immunosuppressed patients (Pinto et al., 2014). PML has been developed due to use of some newly immunosuppresive drugs including natalizumab (Monoclonal antibody against α4β1 and α4β7 integrins) for treatment of multiple sclerosis (MS) and Crohn’s disease ( Berger et al., 2017; Van et al., 2005). It has been reported the treatment of MS patients with rituximab (Monoclonal antibody against CD20) did not develop PML (Asztely et al., 2015). However, some cases of PML have been reported when rituximab was used in the patients with rheumatoid arthritis (Carson et al., 2009). Recently anti-CD20 antibody, ocrelizumab, has been approved by the FDA for relapsing-remitting MS (RRMS) and primary progressive MS (Elizabeth et al., 2018). Fingolimod is a sphingosine 1-phosphate receptor modulator approved for the treatment of relapsing forms of MS (Kappos et al., 2010). Progressive multifocal leukoencephalopathy was developed in patients with multiple sclerosis (MS) after fingolimod treatment (Berger et al., 2018). At this point of time, unfortunately, there is no established treatment for PML. 

In conclusion, in the present study 6.09% of urine samples were tested positive for JCV DNA. The results of sequencing and phylogenetic tree revealed that the isolated JCV DNA were genotype 3A. While 12.8% of urine samples were positive for BKV DNA. The detected BKV DNA was genotype III. With the respect to life threatening diseases due to BKV and JCV, the screening BKV DNA and JCV DNA should be implemented for the patients with autoimmune diseases /organ recipient/cancer/ multiple sclerosis (MS), prior to immunosuppression therapy or immunomodulatory agents treatment.
